# Novel virulence, antibiotic resistance and toxin gene-specific PCR-based assays for rapid pathogenicity assessment of *Arcobacter faecis* and *Arcobacter lanthieri*

**DOI:** 10.1186/s12866-018-1357-7

**Published:** 2019-01-11

**Authors:** Matthew Zambri, Michel Cloutier, Zaky Adam, David R. Lapen, Graham Wilkes, Mark Sunohara, Edward Topp, Guylaine Talbot, Izhar U. H. Khan

**Affiliations:** 1 Ottawa Research and Development Centre (ORDC), Agriculture and Agri-Food Canada, 960 Carling Ave., Ottawa, Ontario K1A 0C6 Canada; 2 Department of Biology, McMaster University, Hamilton, Ontario L8S 4L8 Canada; 3 School of Information Technology and Engineering, University of Ottawa, Ottawa, Ontario K1N 6N5 Canada; 4 London Research and Development Centre (LRDC), Agriculture and Agri-Food Canada, London, Ontario N5V 4T3 Canada; 5 Sherbrooke Research and Development Centre (SRDC), Agriculture and Agri-Food Canada, Sherbrooke, Quebec, J1M 0C8 Canada

**Keywords:** Mono- and multiplex PCR, *Arcobacter faecis*, *A. lanthieri*, Pathogenicity, Virulence, Antibiotic resistance, Toxins

## Abstract

**Background:**

*Arcobacter faecis* and *A. lanthieri* are two newly classified species of genus *Arcobacter*. The prevalence and distribution of virulence, antibiotic resistance and toxin (VAT) genes in these species are required to assess their potential pathogenic health impacts to humans and animals. This study (i) developed species- and gene-specific primer pairs for the detection of six virulence, two antibiotic resistance, and three toxin genes in two target species; (ii) optimized eight single-tube multiplex and three monoplex PCR protocols using the newly developed species- and gene-specific primers; and (iii) conducted specificity and sensitivity evaluations as well as validation of eleven mono- and multiplex PCR assays by testing *A. faecis* (*n*= 29) and *A. lanthieri* (*n*= 10) strains isolated from various fecal and agricultural water sources to determine the prevalence and distribution of VAT genes and assess the degree of pathogenicity within the two species.

**Results:**

Detection of all ten and eleven target VAT genes, and expression of cytolethal distending toxin (*cdt*A, *cdt*B and *cdt*C) genes in *A. faecis* and *A. lanthieri* reference strains with high frequency in field isolates suggest that they are potentially pathogenic strains. These findings indicate that these two species can pose a health risk to humans and animals.

**Conclusions:**

The study results show that the developed mono- and multiplex PCR (mPCR) assays are simple, rapid, reliable and sensitive for the simultaneous assessment of the potential pathogenicity and antibiotic resistance profiling of *tet*(O) and *tet*(W) genes in these two newly discovered species. Also, these assays can be useful in diagnostic and analytical laboratories to determine the pathotypes and assessment of the virulence and toxin factors associated to human and animal infections.

**Electronic supplementary material:**

The online version of this article (10.1186/s12866-018-1357-7) contains supplementary material, which is available to authorized users.

## Highlights

1. Species- and gene-specific mPCR assays were  developed, optimized and validated for *A. faecis* and *A. lanthieri*
2. Virulence, antibiotic resistance and toxin genes were rapidly detected in field isolates
3. Evaluation of Pathogenicity of *A. faecis* and *A. lanthieri* strains

## Introduction

The genus *Arcobacter* was proposed in 1991 by Vandamme et al. [1], separating it from genus *Campylobacter*. It was further amended and enlarged to include a total of over 27 species, with the reclassification of other *Arcobacter* spp. isolated from various human, animal and environmental sources [2–4]. The *Arcobacter* spp., particularly *A. butzleri*, *A. cryaerophilus*, *A. skirrowii*, and *A. cibarius*, are of interest to research groups because of their prevalence in bovine, porcine, poultry and shellfish [5, 6]. These species have been considered as emerging food- and waterborne zoonotic pathogens [7]. *Arcobacter* spp. have been associated with various illnesses such as gastroenteritis, bacteremia, and sepsis in humans, mastitis, diarrhea, abortion, and other reproductive disorders in animals [8]. Infection caused by the genus *Arcobacter* spp. is still considered as a low public health risk because of misidentification by inappropriate detection and typing methods of both *Campylobacter* and *Arcobacter* spp., which result in underestimation of the true incidence of *Arcobacter* infection [9]. Moreover, there is a limited knowledge of key mechanisms (e.g., adhesion, invasion, and cytotoxicity capacity) and potential virulence and toxin factors of *Arcobacter* spp. [6, 10, 11]. The pathogenesis of bacterial infection depends on enterotoxins, adherence and colonization factors as well as invasiveness and penetrability characteristics where pathogens causing gastrointestinal infection either attach to the surface of epithelial cells or invade the intestinal epithelial cells and replicate in the intestinal lumen and produce a systemic disease [12].

Among several *Arcobacter* spp., *A. faecis* and *A. lanthieri* are two recently discovered species within the genus *Arcobacter* which were recovered from human waste septic tank and livestock manure, respectively [4, 13]. The two species were distinguished with other *Arcobacter* spp. through polyphasic approaches that examined several genotypic and phenotypic aspects of *A. faecis* (LMG 28516^T^) and *A. lanthieri* (LMG 28519^T^) reference strains [4, 13]. Through the genetic analysis performed on the multiple housekeeping gene sequences, it was reported that the two novel species were most closely related to each other as well as to the *A. cibarius*, *A. butzleri*, *A. cryaerophilus* and *A. skirrowii*. Since these two *Arcobacter* spp. have been isolated from human and animal fecal sources, it is important to investigate the degree of pathogenicity of these species that can aid in identifying their potential relevance to human and animal infections.

Studies have previously evaluated the occurrence and potential role of virulence-associated genes (VAGs) in the pathogenesis of human and animal infections by the *Arcobacter* spp. [10, 14]. Other studies have indicated that several VAGs are prevalent in the genus *Arcobacter* [14–16] which suggests that several of the *Arcobacter* spp. potentially possess the ability to pose a significant risk to human health. Among six virulence genes, *cad*F gene encodes for an integral membrane protein that serves as an adhesion factor which allows the bacteria to bind to the fibronectin proteins found in the extracellular matrix of the host’s intestinal epithelial cells, and is also responsible for activating GTPases of bacterial cells, thus inducing their internalization [17]. The *cia*B gene encodes for the *Campylobacter* invasion antigen B [18], whereas *irg*A gene encodes for an iron-regulated outer membrane protein. However, *mvi*N gene encodes for an essential protein that is involved in peptidoglycan and cellular membrane synthesis and *pld*A gene encodes the outer membrane phospholipase A. In addition, *tyl*A encodes for the exotoxin hemolysin A [19].

On the other hand, cytolethal distending toxin (*cdt*) found in Gram-negative bacteria that has been shown to stall growth of eukaryotic cells in either the G0/G1 or G2/M phases of the cell cycle which inevitably results in cell death. The *cdt* toxin functions as a heterotrimer of proteins encoded by three separate genes (*cdt*A, *cdt*B, and *cdt*C) that have been recognized for the expression of cytotoxin production [20, 21]. Previous studies have reported the distinct absence of *cdt* genes from *Arcobacter* spp. isolated from various sources as well as a genome sequence of *A. butzleri* strain [19, 22]. Interestingly, it has been determined that the *A. faecis* reference strain contained only sequences for the *cdt*A and *cdt*C genes, compared to the *A. lanthieri* that revealed sequences for *cdt*A, *cdt*B and *cdt*C genes, respectively [23, 24].

In addition to the pathogenicity aspect, antimicrobial resistance is a serious and increasing threat to human health since their use for growth promotion or treatment of livestock, poultry and aquaculture has a direct impact on this problem. Among several antimicrobial agents, tetracyclines are still considered a clinically relevant antibiotic with limited use due to dissemination of tolerance and resistance determinants [25]. However, tetracyclines are frequently used in livestock, poultry and aquaculture as therapeutic, prophylactic or growth promotors worldwide with significantly high usage in countries such as China and India compared to United States and Europe due to the ban of tetracycline and other antibiotics as growth promoters which enhances the emergence of antibiotic resistant strains, allergic reactions in humans and animals, and changes in environmental microflora and bacterial populations among other detrimental effects [26–30]. Studies have reported high resistance of *A. cryaerophilus* to tetracycline [31] compared to *A. butzleri* that has shown high susceptibility to tetracycline and strong and widespread resistance to several other antibiotics [32–34]. Tetracycline acts to prevent the synthesis of bacterial proteins by binding to the prokaryotic 30S ribosomal subunit, thus inhibiting initiation of translation [35, 36]. This resistance is due to different tetracycline resistance (*tet*) genes [37]. Of more than 40 genes encoding tetracycline resistance (*tet-*genes) that have been characterized, *tet*(O) and *tet*(W) genes confer ribosomal protection from the inhibiting effect of tetracycline [36, 38]. Therefore, *tet*(O) and *tet*(W) genes were specifically selected for this study as they appear to be promiscuous in environmental organisms through different transfer mechanisms [39, 40], which makes them suitable candidates for understanding the degree of prevalence, distribution and further monitoring of other antibiotic resistance genes in these two species. In order to understand and identify whether these isolates are potentially pathogenic, several virulence, antibiotic and toxin (VAT) genes were identified in both *A. faecis* and *A. lanthieri* reference strains [23, 24] by whole-genome sequence analysis.

The present study was undertaken to determine the prevalence of these genes within the fecal matter and agricultural environment based on development and optimization of a total of eleven (three monoplex and eight multiplex) PCR assays using six virulence (*cad*F, *cia*B, *irg*A, *mvi*N, *pld*A and *tyl*A), three cytolethal distending toxin (*cdt*A, *cdt*B and *cdt*C) and two tetracycline [*tet*(O) and *tet*(W)] genes. The assays were further validated to evaluate the degree of pathogenicity using reference strains and culture isolates recovered from various fecal sources and agricultural surface water samples.

## Materials and methods

### Reference strains and *Arcobacter* field culture isolation 

For this study, a total of 17 different *Arcobacter* spp. along with 25 other bacterial reference species and strains isolated from various sources were used to test the newly developed assays (Table 1). The reference strains were grown on selective culture media under specific culture conditions.

**Table 1:** List of reference strains of *Arcobacter* and other bacterial species used in this study

Sr. #	Species	Source	Strain ID
1	*Arcobacter bivalviorum*	Shellfish	LMG 26154
2	*A. butzleri*	Human diarrheic stool	ATCC 49616
3	*A. cibarius*	Broiler carcasses	LMG 21996
4	*A. cryaerophilus*	Bovine aborted fetus	NCTC 11885
5	*A. defluvii*	Sewage	LMG25694
6	*A. ellisii*	Mussels	LMG 26115
7	*A. faecis*	Human septic tank	LMG 28519
8	*A. halophilus*	Hypersaline lagoon	ATCC BAA-1022
9	*A. lanthieri*	Pig manure	LMG 28516
10	*A. marinus*	Mix seawater, starfish and seaweed	LMG 25770
11	*A. molluscorum*	Mussels and oysters	LMG 25693
12	*A. mytili*	Mussels	LMG 24559
13	*A. nitrofigilis*	Roots	ATCC 33309
14	*A. skirrowii*	Lamb feces	ATCC 51322
15	*A. thereius*	Organs of aborted porcine	LMG 24486
16	*A. trophiarum*	Feces of fattening pigs	LMG 25534
17	*A. venerupis*	Shellfish	LMG 26156
18	*Aeromonas allosaccharophila *	Diseased elvers	ATCC 51208
19	*A. bestiarum*	Infected fish	ATCC 51108
20	*A. caviae*	Epizootic of young guinea pigs	ATCC 15468
21	*A. hydrophila*	Ditch water	ATCC 13444
22	*A. jandaei*	Human feces	ATCC 49568
23	*A. media*	Marine fish	CDC 0435-84
24	*A. popoffi*	Drinking water production plant	BAA-243
25	*A. salmonicida*	Freshwater	CDC 0434-84
26	*A. schubertii*	Skin	ATCC 43700
27	*A. sobria*	Sludge	ATCC 35994
28	*A. trota*	Human feces	ATCC 49658
29	*A. veronii*	Red-leg frog	ATCC 9071
30	*A. bv. veronii*	Amputation Wound	ATCC 35625
31	*Campylobacter jejuni*	Human feces	ATCC 33291
32	*C. coli*	Swine	ATCC 43136
33	*C. lari*	Human feces	ATCC 43675
34	*C. helveticus*	Cat	ATCC 51210
35	*C. fetus subsp. fetus*	Blood	ATCC 15296
36	*C. hyointestinalis*	Intestine of swine	ATCC 35217
37	*Escherichia coli* O157:H7	Environmental isolate	-
38	*E. coli*	Canine	ATCC 35218
39	*Pseudomonas shigelloides*	Environmental isolate	-
40	*Salmonella enterica subsp. arizonae*	-	ATC C 13314
41	*S. enterica subps. diarizonae*	-	ATCC 12325
42	*S. enterica subsp. houtenae*	-	ATCC 29932

In addition, 29 *A. faecis* and ten *A. lanthieri* culture strains isolated from various environment including agricultural surface water and fecal sources were tested for assessment, validation and application of the three monoplex and eight multiplex PCR assays. *Arcobacter* cultures were recovered from human and animal fecal and agricultural water sources using a method previously described by Whiteduck-Léveillée et al. [13]. Briefly, the fecal samples were collected under aseptic conditions using sterile spatula and containers, and 1 g of fecal sample was ten-fold serially diluted by mixing in 9 mL of peptone water. The suspension (100 μL) was directly plated on Arcobacter Selective Isolation Agar (ASIA) (Oxoid, Nepean, ON) containing antimicrobial (fluorouracil, amphotericin-B, cefoperazone, novobiocin and trimethoprim) agents. However, the recovery of *Arcobacter* cultures from agricultural surface water samples, collected in sterile polypropylene bottles, was carried out by processing 500 mL of water through 0.22 μm sterile nitrocellulose filter using a vacuum-filtration system. The filters were placed on ASIA plate and incubated at 30°C under microaerophilic condition (85% N_2_, 10% CO_2_ and 5% O_2_) for 3 to 6 days. The presumptive *Arcobacter* culture isolates were, initially, selected based on conventional cultural characteristics (growth pattern and colony morphology) and Gram staining reactions. Further confirmation was carried out by *Arcobacter* genus- and species- specific PCR assays [41, 42].

### Tetracycline susceptibility test

For pheno- and genotypic analysis, susceptibility of *A. faecis* and *A. lanthieri* strains to tetracycline was tested on Mueller-Hinton agar (Oxoid) using the agar dilution method according to CLSI [43]. The plates were incubated for 3 days in a microaerophilic condition at 30°C. Results were interpreted in accordance with the CSLI guidelines [44].

### Nucleic acid extraction

For assay development, optimization and validation, the nucleic acid was extracted from reference strains and *Arcobacter* culture isolates recovered from surface water and fecal samples according to previously described boiling method [45]. Briefly, a single purified colony was suspended and gently mixed in a 100 μL 1xTE (pH 8.0) buffer. The cells were boiled (10 min.) and centrifuged (1 min.) at high speed. The purity and concentration of extracted DNA was determined by NanoDrop (ND-1000) spectrophotometer and agarose gel electrophoresis.

### Gene-specific primers design, development and optimization of mono- and multiplex PCR assays for *A. faecis*

Based on a comprehensive whole-genome sequence analysis of *A. faecis* LMG 28519 reference strain, six virulence (*cad*F, *cia*B *irg*A, *mvi*N, *pld*A and *tyl*A), two antibiotic resistance [*tet*(O) and *tet*(W)] and two cytolethal distending toxin (*cdt*A and *cdt*C) genes were identified. After alignments of these VAT gene sequences with other *Arcobacter* reference species sequences available in the GenBank database, a total of ten sets of VAT gene-specific oligonucleotide primer pairs for *A. faecis*, ranging from 20 to 28-mers, were designed. The ten pairs of primers, with their predicted fragment sizes, were used in one monoplex and four mPCR protocols as listed in Table 2. In order to check the specificity of the designed primers, initially, monoplex PCR assays were performed using only the forward and the reverse primer for detection of individual target gene. The PCR amplification reaction for each individual assay was carried out in the Mastercycler Gradient PCR system (Eppendorf, Hauppauge, NY), with a 25 μL reaction mixture containing 10-50 ng of *A. faecis* LMG 28519 DNA template, 1 U of *Ex-Taq* DNA polymerase and the compatible PCR reagents, including 1× buffer with MgCl_2_, 200 μM each of the dNTPs (Fisher Scientific, Nepean, ON), 0.1 μM of each set of the forward and reverse primer pair by adjusting volume to 25 μl with sterile distilled water in each monoplex PCR assay. The PCR reactions were conducted with an initial template denaturation (94°C for 3 min) followed by 35 cycles of amplification (denaturation at 94°C for 30 s, annealing ranging from 54 to 59°C for 30 s and extension at 72°C for 30 s) ending with a 5 min extension at 72°C for individual target genes. For all PCR reactions, *A. faecis* LMG 28519 as positive and *A. butzleri* ATCC 49616, *A. cibarius* LMG 21996 and *A. lanthieri* LMG 28516 reference strains as negative controls were used. The specificity of all ten primer sets was further evaluated individually using 13 *Arcobacter* spp. and 25 other bacterial reference species and strains (Table 1).

**Table 2:** List of oligonucleotide primer pairs and PCR protocols designed and optimized for the detection of VAT genes in *A. faecis*

Target VAT Gene	Oligonucleotide Primer Sequences (5’-3’)	Amplicon Size (bp)	Primer Concentration (μM)	Annealing Temperature (°C)
Assay 1				
*mvi*N	mviN F: TTC TTT GCA GCA ACA TTG GG	180	0.4	57
	mviN R: TGC TAC CAT AGG AAA TAG GGC		0.4	
*irg*A	irgA F: CTG GAC AGT ATG AAG GAA ACC C	105	0.4	
	irgA R: TTG CTT GAG TCC ATA ACA ACC A		0.4	
Assay 2				
*cad*F	cadF F: ATG GTG CAT TCG GAA ACT ACG G	192	0.1	60
	cadF R: TTG GAG CTG GAG CAG GAA CTA		0.1	
*tyl*A	tylA F: TAG AAA CAA AGC AAG TGA ACT C	116	0.2	
	tylA R: CCT CTT CAA GTA GCT CTA TAT T		0.2	
Assay 3				
*cdt*A	cdtA F: TGT AGC CGA TGA ACT TAG TGA AGT AGA G	274	0.4	59
	cdtA R: CCC AAC TGT TGC TTG TCC CAT TA		0.4	
*cia*B	ciaB F: AAG CAG TTG CCC TAG AGT GG	196	0.4	
	ciaB R: AGT GCT GGT CGT CCA ACA TAA		0.4	
*pld*A	pldA F: GTG CTG CTG AAT TTA ACT GG	145	0.4	
	pldA R: GCA ACA CCT ATT CCT ACA TTT G		0.4	
Assay 4				
*cdt*C	cdtC F: AAG CAG AGG GTG AAA TAG CC	210	0.8	56
	cdtC R: GAT TAG CAA ACT GTC CAC CAA ATA C		0.8	
*tet*(W)	tet(W) F: ACA TCA TTG ATA CTC CAG GTC ACG	120	0.2	
	tet(W) R: TTT CAC TTT GTG GTT GAA CCC CTC		0.2	
Assay 5				
*tet*(O)	tetO F: GGA GGG GTT CAA CCA C AA AG	88	0.1	54
	tetO R: CTA TGT AAA TAA AAT GGA TAG		0.1	

In order to detect VAT genes in a more rapid and robust manner, four mPCR assays were optimized by combining and standardizing PCR protocols using two or three VAT gene-specific oligonucleotide primers. Each assay was designed such that the combination of the two or three primer pairs distinctively differed by a minimum of 50 bp in size in order to distinctively resolve PCR fragments on an agarose gel electrophoresis (Table 2). Based on the monoplex PCR results, mPCR assays were further optimized where various parameters particularly concentrations of primers (0.1 to 1.0 μM), *Ex-Taq* DNA polymerase (0.6 to 2 U), dNTPs (100 to 200 μM each of the four dNTPs), buffer strength (1.0× to 2.0×) and concentration (0.1-0.5 μM) were tested. The simultaneous gene amplifications were performed in a reaction volume of 25 μl consisting of variable (1 to 100 ng μL^-1^) DNA concentrations. Four PCR tubes containing two or three sets of primer pairs for the simultaneous detection of nine target VAT genes were processed with an initial denaturation at 94°C for 2 min followed by 35 cycles (except 30 cycles for *cad*F and *tyl*A genes) of amplification including denaturation at 94°C for 30 s, different annealing temperatures (ranging from 54 to 60°C) for each mPCR assay (Table 2) and extension at 72°C for 30 s ended with a 5 min final extension at 72°C.

The expected amplicon sizes were small; therefore, each mono- and multiplex PCR reaction was electrophoresed on a 2.5% agarose gel matrix (Fisher Scientific). A 100 bp DNA size marker (Life Technologies, Grand Island, NY) was used to confirm the expected amplicon sizes of each target gene. The agarose gels were stained (ethidium bromide 0.5μg mL^-1^), scanned and photographed using an Alpha Imager (Fisher Scientific) gel documentation system.

### Gene-specific primers design and optimization of mono- and multiplex PCR assays for *A. lanthieri*

For *A. lanthieri*, a total of 11 including six virulence (*cad*F, *cia*B, *irg*A, *mvi*N, *pld*A, and *tyl*A), two antibiotic resistance [*tet*(O) and *tet*(W)] and three cytolethal distending toxin (*cdt*A, *cdt*B, and *cdt*C) genes were identified based on genome sequence analysis of *A. lanthieri* LMG 28516 reference strain. Each gene sequence was further aligned with the sequences of other *Arcobacter* reference species available in the GenBank database. For mono- and multiplex PCR assays, 11 *A. lanthieri* gene-specific oligonucleotide primer sets (ranging from 20 to 24-mers) were designed from variable regions of each gene with a minimum of 50 bp for easy differentiation of amplicons on an agarose gel electrophoresis analysis (Table 3). The specificity of each assay was individually performed as monoplex PCR using *A. lanthieri* LMG 28516 reference strain, several *Arcobacter* spp. and other bacterial species and strains as positive and negative controls, respectively.

**Table 3:** List of oligonucleotide primer pairs and protocols designed for the detection of VAT genes in *A. lanthieri* strains

Target VAT Gene	Oligonucleotide Primer Sequences (5’-3’)	Amplicon size (bp)	Primer Concentration (μM)	Annealing Temperature (°C)
Assay 1				
*cdt*B	cdtB F: GCA AAA GGT GAT TGG GCT CC	303	0.4	56
	cdtB R: TCC TCC AGC TCC TTG AAC AC		0.4	
*cad*F	cadF F: TCC AAC TCC AGT TGC TGC TC	243	0.4	
	cadF R: TGT CCT TCG ATG TCA GCT TTC		0.4	
*irg*A	irgA F: AGA GCT GTT GGT TGG GAT GG	186	0.4	
	irgA R: TGC ATT TGC TCT TGT AGG GT		0.4	
Assay 2				
*cdt*C	cdtC F: GAT GAA TCC ACC AGA AAT AGA G	196	0.3	57
	cdtC R: TTT GGG ATC AAG AGT ATA AAG TTC		0.3	
*pld*A	pldA F: TGC TCC ATT TAG AGA AAC TAA C	132	0.1	
	pldA R: GAA CGA GAT TCT TCA CCA TCT T		0.1	
Assay 3				
*cdt*A	cdtA F: CAG GAA TAG ATC TCG CTA CAA ATG	220	0.3	55
	cdtA R: TTT GGT AGA AGA GGA AGT TCA TTG		0.3	
*mvi*N	mviN F: ACC TTT GGT TCT TCA ACT TTA C	170	0.4	
	mviN R: CGT GCT ACC ATA GGA AAT AGG		0.4	
Assay 4				
*cia*B	ciaB F: GAT AGA TGC TAT TCT GCT CTT G	207	0.2	60
	ciaB R: ATC TTC ACT AAA TGC TAC TAT T		0.2	
*tyl*A	tylA F: GAC ATT GTA ACA TGT GAT GTA TCT T	125	0.1	
	tylA R: TTT ACA TTT GTT CCC ACT TCA AA		0.1	
Assay 5				
*tet*(O)	tetO F: TCA TAC ACT TAT ACA CTT GAT GC	94	0.2	62
	tetO R: CTC TAT ATT CAT TTG CAA CAT CAA CT		0.2	
Assay 6				
*tet*(W)	tetW F: AAT CAT ACA CTT ATA CAC TTG ATG CAC	197	0.1	60
	tetW R: TTT AAT TCC TTC AAC TAT CTC TTC TTC		0.1	

Based on gene-specific amplification results of each developed monoplex PCR assay, four mPCR assays were further optimized using a combination of two or three sets of primer pairs of six virulence and three toxin genes. As mentioned in the preceding section, several parameters were investigated to optimize mPCR protocols including primer concentrations (0.1 to 1.0 μM), *Ex-Taq* DNA polymerase (0.5 to 2 U), dNTPs (100 to 200 μM each of the four dNTPs) and buffer strength (1.0× to 2.0×) along with Bovine Serum Albumin (BSA) concentration (0.1 to 0.3 μM) were tested in order to obtain equal intensity of the amplified mPCR products. The mPCR assays were performed in 25 μL reactions with a minimum DNA concentration of 10 ng μL^-1^. Each mPCR assay, which incorporates the detection of either two or three virulence and/or toxin genes, consisted of a 35-cycle PCR program with an initial denaturation cycle of 94°C for 2 min, intermediate intra-cycle steps of 30 s each for denaturation at 94°C along with annealing where temperatures (ranging from 55 to 62°C) varied from assay to assay (Table 3) and extension at 72°C ended with a final elongation step of 5 min at 72°C. 

Each mono- and multiplex PCR product was analyzed on an agarose gel electrophoresis, visualized on an ultraviolet transilluminator and photographed as described above.

### Sensitivity and specificity assessment of mono- and multiplex PCR assays

The sensitivity of eleven optimized mono- and multiplex PCR assays was evaluated with representation of each target VAT gene specific for *A. faecis* and *A. lanthieri*. The purified DNA of each target species reference strain was quantified spectrophotometrically at 260 nm optical density, and ten-fold serially diluted (ranging from 100 ng μL^-1^ to 10 pg μL^-1^) corresponding to 10^5^ to 10^1^ CFU mL^- 1^ were prepared in sterile water. The detection limits were determined for each target species and gene by the mono- and multiplex PCR assays. In order to avoid any cross-reaction of our species- and gene-specific primers with *Arcobacter* species, other than *A. faecis* and *A. lanthieri*, and 40 other bacterial reference species and strains along with negative controls (PCR master mix without DNA template and water) were also included in each assay.

### Evaluation and validation of specificity of developed assays

In order to evaluate the performance in terms of reproducibility and validity of the designed primers and PCR conditions for a total of three mono- and eight multiplex PCR assays, 29 *A. faecis* and ten *A. lanthieri* strains from various human and animal fecal sources and agricultural surface water samples were used for this study. The isolates were previously tested using standard conventional microbiology procedures and further confirmed by genus- and species-specific PCR assays as described above. Furthermore, the specificity of amplified products of 21 target VAT genes were evaluated by sequencing PCR amplicons of two of each target species isolated from fecal and water samples along with reference strains were purified using a QIAquick gel extraction kit (Qiagen, USA). The purified PCR products were sequenced using an ABI 3130xL Genetic Analyzer (Applied Biosystems) according to the manufacturer’s recommendations. The data was analyzed against the global database using a BLAST search engine for species- and gene-level confirmation of positive isolates.

### RNA extraction and *cdt* gene expression analysis

In order to examine the transcriptional expression of the *cdt* (*cdt*A, *cdt*B and *cdt*C) genes among *A. faecis* and *A. lanthieri* isolates, total RNA was extracted by using NucleoSpin RNA kit (Macherey Nagel, D-Mark Bioscience, Germany) according to the manufacturer’s instructions. RNA was suspended in an appropriate amount of RNase-free water and RNA concentrations estimated by Qubit 3.0 fluorometer (Thermo Fisher Scientific, Waltham, MA, USA). Reverse transcriptase PCR (RT-PCR) was carried out using iScript gDNA Clear cDNA synthesis kit (BioRad, USA) according to the manufacturer’s instructions. RT-PCR primers listed in Tables 2 and 3 were used to amplify *cdt*A, *cdt*B and *cdt*C genes. PCR reactions were performed on the cDNA template after the addition of *Taq* DNA polymerase, deoxynucleoside triphosphates, 1× buffer with MgCl_2_ and each set of *cdt* gene primers with the incubation conditions of 95°C for 2 min followed by 35 cycles at 95°C for 30 s, 56 to 59°C for 30 s and 72°C for 30 s with a final incubation at 72°C for 5 min. PCR products were run on a 2% agarose gel and visualized.

## Results

### Specificity and optimization of species- and gene-specific primers and protocols in monoplex PCR assays

Initially, the specificity of 21 VAT gene primers and optimization of PCR protocols for *A. faecis* and *A. lanthieri* were tested with their corresponding reference DNA by monoplex PCR (Tables 2 and 3), and confirmed the expected amplicon sizes by agarose gel electrophoresis (data not shown). In this optimization step, all ten and 11 VAT genes investigated were detected in the *A. faecis* and *A. lanthieri* reference strains, respectively. The *A. faecis* primers specifically amplified with expected amplicon sizes for six virulence [*cad*F (192 bp), *cia*B (196 bp), *irg*A (105 bp), *mvi*N (180 bp), *pld*A (145 bp) and *tyl*A (116 bp)], two  *cdt* [*cdt*A (274 bp) and *cdt*C (210 bp)] and two antibiotic resistance [*tet*(O) (88 bp) and *tet*(W) (120bp)] genes, respectively. Similarly, for *A. lanthieri* reference strain, specific PCR products were obtained with expected sizes for six virulence [*cad*F (243 bp), *cia*B (207 bp), *irg*A (186 bp), *mvi*N (170 bp), *pld*A (132 bp) and *tyl*A (125 bp)], three cytolethal distending toxin [*cdt*A (220 bp), *cdt*B (303), and *cdt*C (196 bp)] and two antibiotic resistance [*tet*(O) and *tet*(W)] genes with typical amplicon sizes of 94 and 197 bp, respectively. The specificity of the developed primers and monoplex PCR protocols for each target species and VAT genes was also tested on 15 *Arcobacter* and other bacterial reference strain DNA templates (Table 1). The results showed that all assays had specificities only for *A. faecis* and *A. lanthieri* reference strains tested.

### Development and optimization of mPCR assays

**Fig. 1:**
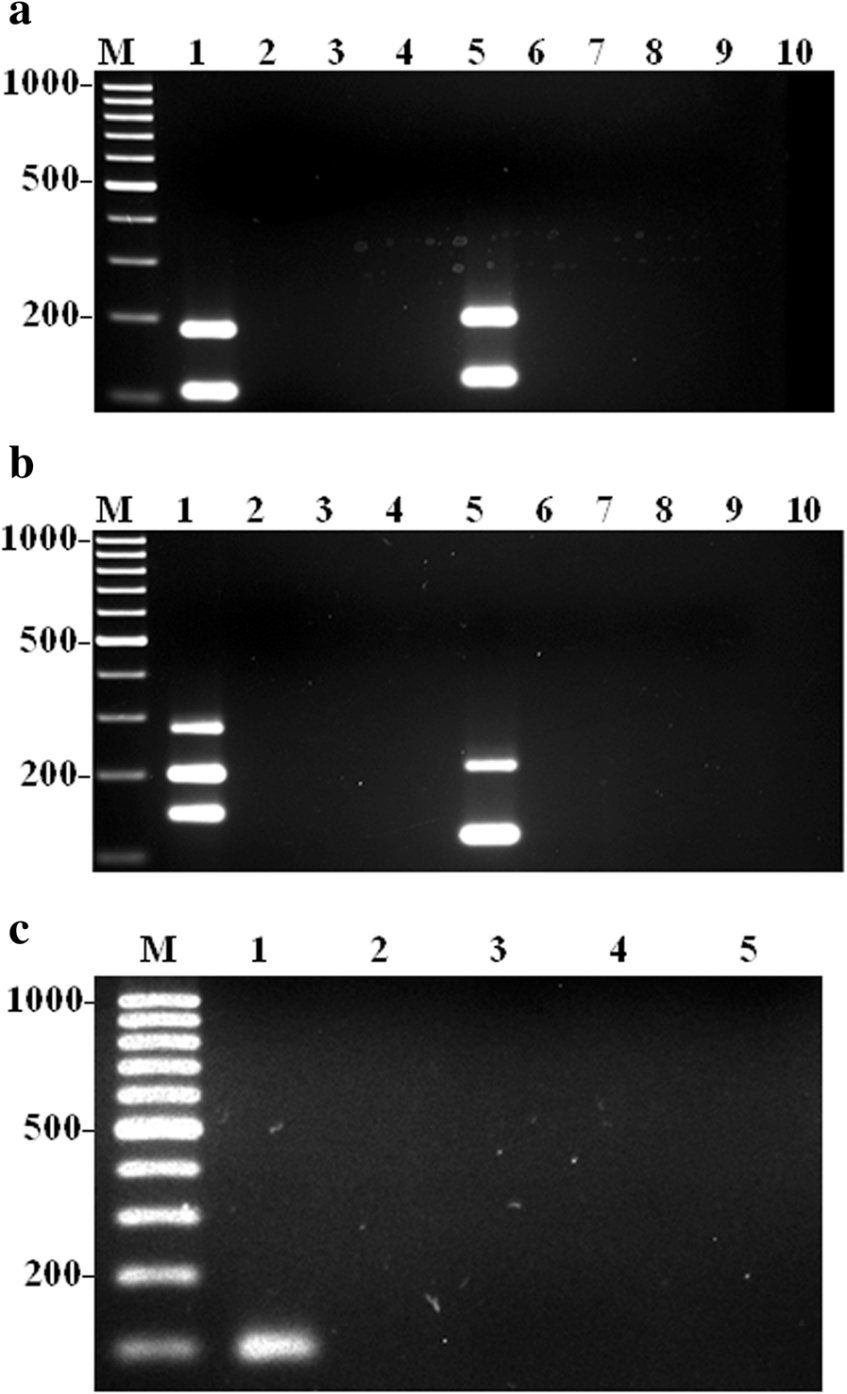
Typical amplicons for gene-specific mPCR assay developed for the detection of VAT genes in *A. faecis*. Panel **a** Lane 1: *mvi*N (180 bp) and *irg*A (105 bp); and Lane 5: *cad*F (192 bp) and *tyl*A (116 bp) genes; Panel **b** Lane 1: *cdt*A (274 bp), *cia*B (196 bp) and *pld*A (145 bp); and Lane 5: *cdt*C (210 bp) and *tet*(W) (120 bp) and; Panel **c** Lane 1: *tet*(O) (88 bp) gene. Lanes 1: *A. faecis* LMG 28519 served as control positive; Lanes 2-4 and 6-8: *A. butzleri* ATCC 49616, *A. cibarius* LMG 21996, *A. lanthieri* LMG 28516 and Lanes 5 (Panel **c**), 9&10 (Panels **a** and **b**) served as control negative. Lane M: 1kb DNA marker

**Fig. 2:**
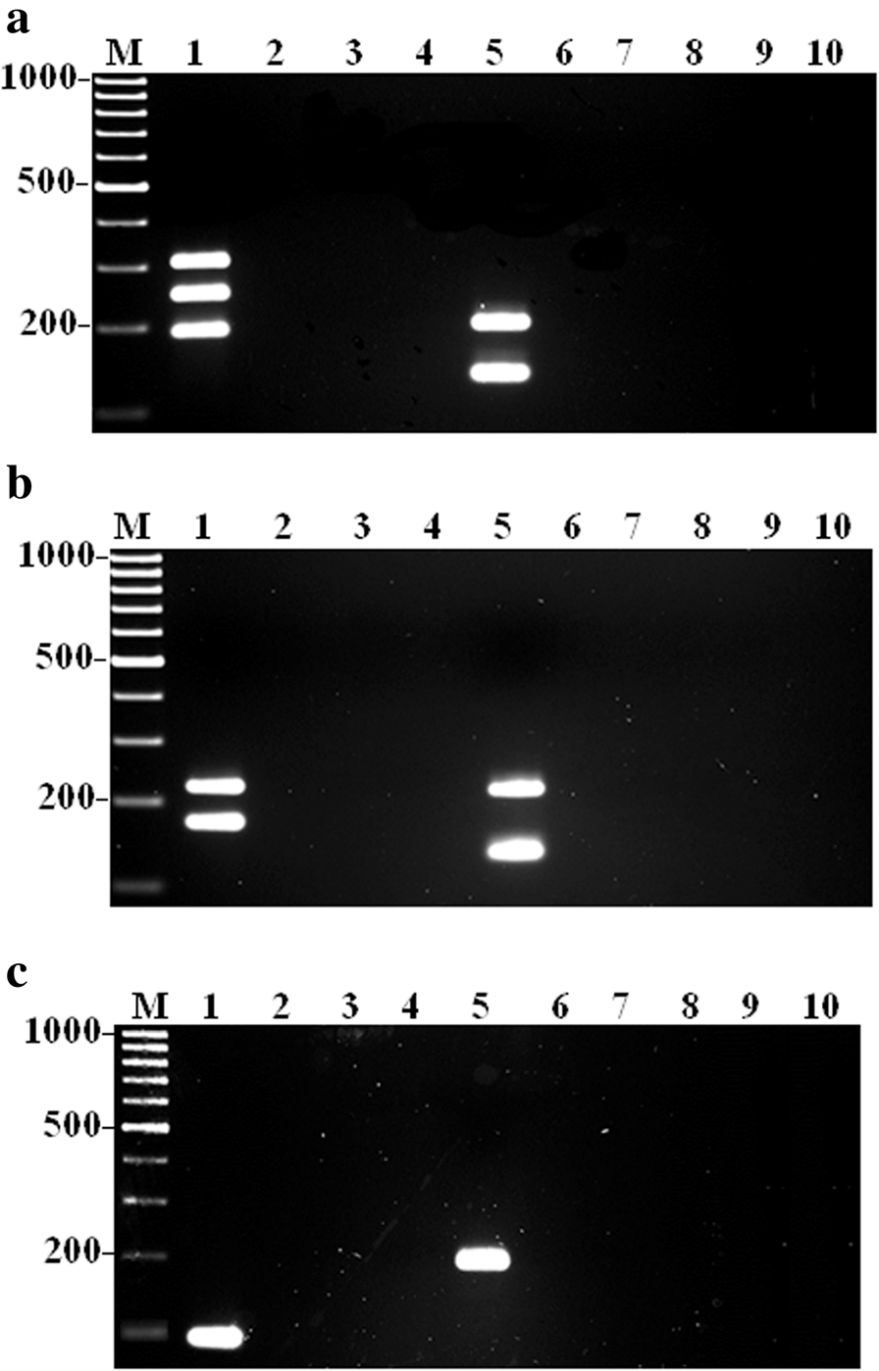
Typical amplicons for each gene-specific mono and multiplex PCR assays developed for the detection of virulence, antibiotic resistance and toxin genes in *A. lanthieri*. Panel **a** Lane 1: *cdt*B (303 bp), *cad*F (243 bp) and *irg*A (186 bp); Lane 5: *cdt*C (196 bp) and *pld*A (132 bp); Panel **b ** Lane 1: *cdt*A (220 bp) and *mvi*N (170 bp); Lane 5: *cia*B (207 bp) and *tyl*A (125 bp); Panel **c** Lane 1: *tet*(O) (94 bp); Lane 5: *tet*(W) (197 bp); Lanes 1&5: *A. lanthieri* LMG 28516 served as control positive; Lanes 2-4 and 6-8: *A. butzleri* ATCC 49616, *A. cibarius* LMG 21996 *A. faecis* LMG 28519 and Lanes 9&10 served as control negative. Lane M: 1kb DNA marker

Furthermore, a total of eight single-tube mPCR assays were developed and optimized for the detection of nine VAT genes in *A. faecis* and *A. lanthieri* reference strains, respectively. However, PCR assays for three antibiotic resitance genes including *tet*(O) for *A. faecis* and*tet*(O) and *tet*(W) for *A. lanthieri* were remain used as monoplex due to the limitation in amplicon sizes and cross-amplification reaction with other genes investigated. To optimize the amplification of all primer pairs in the mPCR assays, initially the optimum annealing temperature for the mPCR reaction was determined. Temperature gradient PCR reactions determined that the appropriate annealing temperature ranged from 54 to 62°C for the mPCR assays. To further optimize each mPCR assay, the manipulation of primer concentration showed effective results. In each monoplex PCR assays, 0.4 μM primer concentration was used; however; to improve differences in the levels and intensity of amplification between different amplicons, the concentration of the primer pairs that amplified more efficiently were optimized using 0.1 to 0.4 μM concentration, while conversely, the concentration of the primer pair that amplified less efficiently were increased to 0.8 μM (Tables 2 and 3). BSA significantly enhanced PCR amplification yield when used at 0.3 μM. In each case, the PCR assays showed no primer-dimer interference or non-specific bands and produced the expected amplicon fragment sizes as obtained in monoplex PCR assays for *A. faecis* including 180 and 105 bp for *mvi*N and *irg*A genes (Assay 1); 192 and 116 bp for *cad*F and *tyl*A genes (Assay 2); 274, 196 and 145 bp for *cdt*A, *cia*B and *pld*A genes (Assay 3); 210 and 120 bp for *cdt*C and *tet*(W) genes (Assay 4); whereas 88 bp for *tet*(O) gene (Assay 5) (Table 2; Fig. 1a, b, and c). On the other hand, each optimized mPCR assay, for *A. lanthieri*, produced same amplicons obtained during optimization of monoplex PCR assays with expected amplicon sizes of 303, 243 and 186 bp for *cdt*B, *cad*F and *irg*A genes (Assay 1); 196 and 132 bp for *cdt*C and *pld*A genes (Assay 2); 220 and 170 bp for *cdt*A and *mvi*N genes (Assay 3); 207 and 125 bp for *cia*B and *tyl*A (Assay 4), whereas 94 bp for *tet*(O) (Assay 5) and 197 bp for *tet*(W) (Assay 6) (Table 2; Fig. 2a, b and c). Each mPCR assay was further evaluated for the specificity of the protocol by using control reference strains of *Arcobacter* spp. The mono- and multiplex PCR products were specifically obtained for each target gene of reference or field strain DNA, which indicates an absence of interference and non-specificity between any of the VAT gene primers or among amplified products.

### Evaluation of developed PCR assays and prevalence of VAT genes

**Fig. 3:**
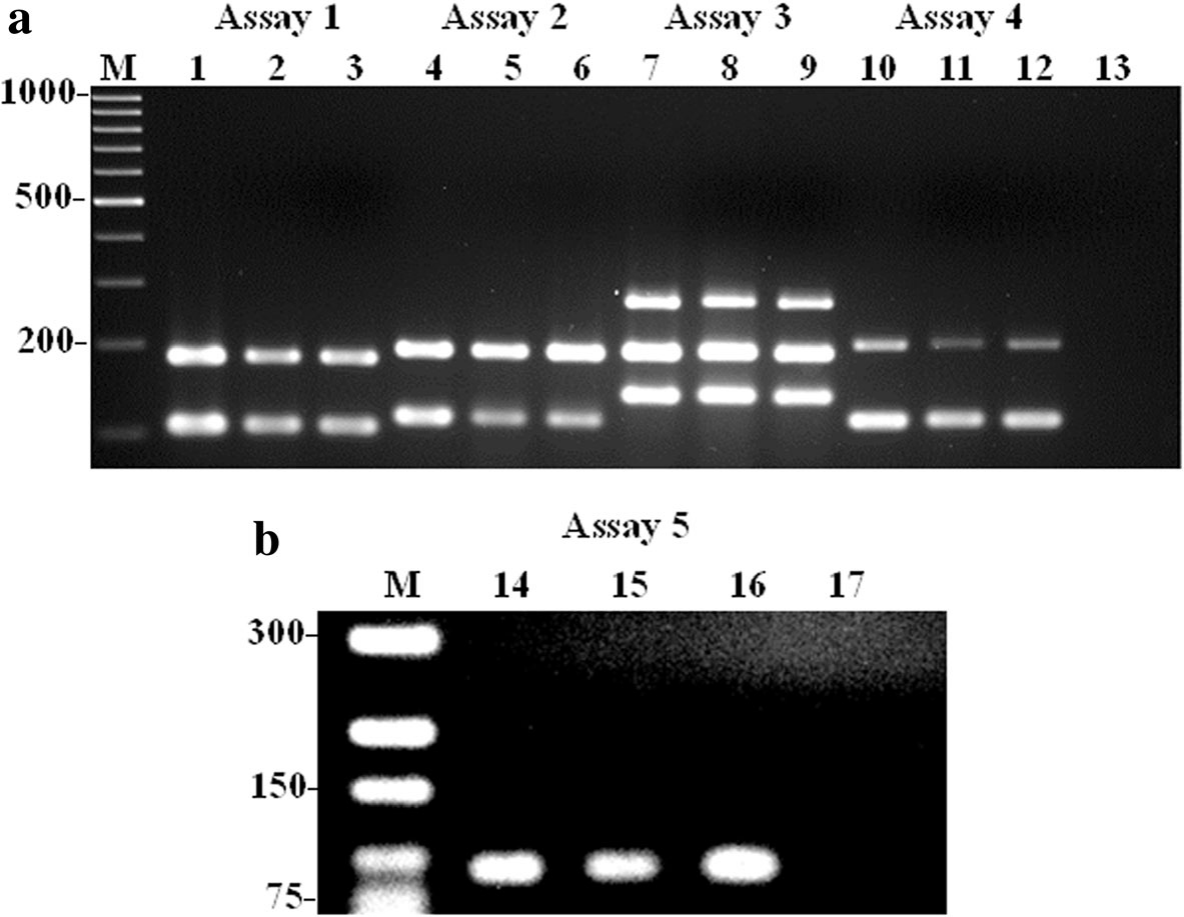
Evaluation and validation of five species-specific mono- and multiplex PCR assays developed for the detection of VAT genes in *A. faecis* field isolates. Panels **a** and **b** Lanes 2-3: *irg*A and *mvi*N genes; Lanes: 5-6 *cad*F and *tyl*A genes; Lanes: 8-9 *cdtA*, *cia*B and *pld*A; Lanes 11-12: *cdt*A and *tet*(W) genes; and Lanes 15-16 *tet*(O) gene. Lanes 1, 4, 7, 10 and 14: Control positive *A. faecis* LMG 28519; Lane 13&17: Control negative; Lane M: 1kb and 300 bp DNA Markers

**Fig. 4:**
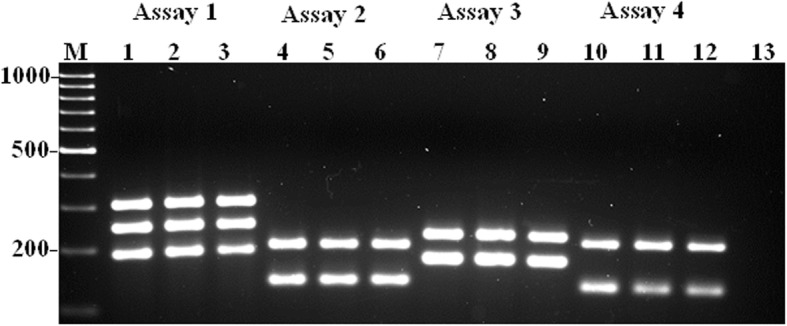
Evaluation and validation of four species-specific mPCR assays developed for the detection of VAT genes in *A. lanthieri* field isolates (Lanes 2-3: *cdt*B, *cad*F, and *irg*A genes; Lanes 5-6: *cdt*C and *pld*A genes; Lanes 8-9: *cdt*A and *mvi*N genes; Lanes 11-12: *cia*B and *tyl*A genes). Lanes 1, 4, 7 and 10: *A. lanthieri* LMG 28516 control positive; Lane 13: Control negative; Lane M: 1kb DNA Marker

**Fig. 5:**
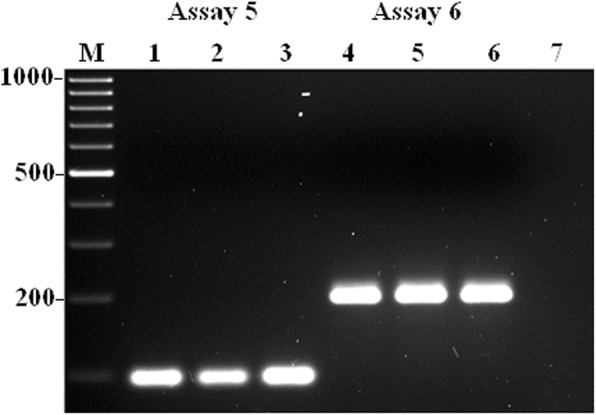
Evaluation and validation of PCR amplicons from each assay developed for the detection of *tet*(O) (Lanes 1-3) and *tet*(W) (Lanes 4-6) genes in *A. lanthieri* field isolates: Lanes 1 and 4: Control positive *A. lanthieri* LMG 28516; Lanes 2-3 and 5-6: field isolates; Lane 7: Control negative; Lane M: 1kb DNA Marker

In order to validate the developed mono- and multiplex PCR assays, and determine the degree of pathogenicity of the *A. faecis* and *A. lanthieri* by investigating the prevalence of VAT genes in field isolates, a total of 29 *A. faecis* cultures isolated from human and animal fecal and agricultural water samples were tested (Fig. 3). Interestingly, of the total ten VAT genes tested, all strains showed positive amplification for all genes at a high frequency ranging from 93 to 100% (Table 4) where *cad*F, *irg*A, *tyl*A, *cdt*C and *tet*(W) genes were detected in all strains. However, of the total ten *A. lanthieri* strains (Figs. 4 and 5), only one strain showed negative amplification reaction for all VAT genes. However, one strain showed negative amplification for *irg*A, *tyl*A, *tet*(O), *cdt*A and *cdt*C genes (Table 4). Similarly, based on the CLSI tetracycline breakpoints, all except one isolate of each species showed resistance to tetracycline which correlates with the *tet*(O) and *tet*(W) gene and species-specific PCR results. In addition, of the total 29 *A. faecis* and 10 *A. lanthieri* isolates, results of the RT-PCR analyzing the expression of *cdt* encoding gene showed detection of *cdt*A (70%; *n*= 20) and *cdt*C (87%, *n*= 25) genes in *A. faecis* compared to *A. lanthieri* isolates where expression of *cdt*A, *cdt*B and *cdt*C genes was detected at a variable rate ranging from 60 to 70%.

**Table 4:** Number (percent) of virulence, antibiotic resistance and toxin (VAT) genes detected in *A. faecis* and *A. lanthieri* strains isolated from agricultural surface water and  various fecal sources

Species	No. of Isolates	Virulence genes						Antibiotic resistance genes		Toxin genes		
		*cad*F	*cia*B	*irg*A	*mvi*N	*pld*A	*tyl*A	*tet*(O)	*tet*(W)	*cdt*A	*cdt*B	*cdt*C
*A. faecis*	29	29 (100)	28 (97)	29 (100)	27 (93)	28 (97)	29 (100)	27 (93)	29 (100)	28 (97)	ND^a^	29 (100)
*A. lanthieri*	10	9 (90)	9 (90)	8 (80)	9 (90)	9 (90)	8 (80)	8 (80)	9 (90)	8 (80)	9 (90)	8 (80)

^a^Gene not detected

The specificity of each targeted gene-specific primer pair of *A. faecis* and *A. lanthieri* was confirmed by sequencing of positive amplicons of reference strains and selected field isolates (Additional file 1: Table S1). The sequences were confirmed by available sequence data analysis where ≥99% sequence homology was observed to the corresponding target species-specific VAT gene.

### Sensitivity of the developed PCR assays

Furthermore, the detection sensitivity of each mono- and multiplex PCR assay developed for a total of 21 genes of two target species was determined and confirmed by examining serially diluted (ten-fold) DNA templates (ranging from 100 ng μL^-1^ to 10 pg μL^-1^) of each target gene primer mixtures. Each targeted gene was amplified with a DNA concentration as low as 10 pg μL^-1^ by observing distinctive multiple PCR products with expected amplicon sizes on a 2.5% agarose gel which suggests that a minimum of 10 pg μL^-1^ of genomic DNA can detect target VAT gene in one of the target species by using this method.

## Discussion

Although *Arcobacter* spp. have been considered as emerging human pathogens, the pathogenicity, toxin production and virulence mechanism still need to be fully elucidated [32]. After the first *A. butzleri* genome sequence, several virulence-associated genes were revealed that showed similarities with other bacteria such as *Campylobacter*, *E. coli* and *Vibrio* spp. [19], while a number of studies reported the prevalence of these genes in *Arcobacter* spp. [6, 10, 14]. These study findings established a cornerstone in identifying *Arcobacter* as an important pathogen that has potential to cause health risk to humans and animals. Of the other available detection techniques, these studies applied PCR assay that has an ability to detect the pathogens and target genes more rapidly and accurately compared to culturing, microscopy, and antigen based tests that have limited detection capacity especially when it comes to detection of a specific gene or pathogenic organism to the species-level. However, mPCR assay can be performed from a single or set of DNA templates rather than analyzing several DNA aliquots for an individual species or gene where the results can be obtained in more efficient and cost effective manner by reducing the possibility of cross-contamination. The major advantage of combining multiple primer pairs together in a single-tube PCR assay reduces the overall quantity of materials used per assay, the amount of time required for high throughput screening, thereby reducing the cost of performing these assays in more robust and rapid manner. However, the major challenges in developing an mPCR assay are primer design, primer concentration, PCR protocol (e.g., annealing and extension time and cycles) and avoiding primer-dimer formation for obtaining an optimal amplification of multiple products for all target species or genes [46]. Formation of primer dimer occurs prior to the first PCR cycle and is typically caused by primers that have complementary overlaps. By choosing a variable terminal sequence at 3’ ends of multiple primers, the formation of stable overlaps can successfully be avoided [47]*.*

With the rapidly evolving technology, genome sequence analysis is becoming common to identify and confirm specific genes for assessing the pathogenicity of a microorganism because of the improved BLAST program and continuous update of genome data. This approach is more convenient and effective e.g., virulence and antibiotic resistance-specific genes were identified in *A. butzleri* and *A. cryaerophilus* by comparative genomics [19, 48]. Researchers have developed mPCR assays for rapid identification of *Arcobacter* spp. [45, 49] as well as analysis of the virulence-associated genes of *Arcobacter* spp. [16]. The *Arcobacter* genus-specific virulence-associated gene-based mono- and multiplex PCR assays have developed and are considered a better target for assessing the pathogenicity of *Arcobacter* spp. isolated from various sources [10, 11, 16, 50]*.* However, these PCR assays were less accurate and failed to amplify all targeted virulence-associated genes in other *Arcobacter* spp. except *A. butzleri* likely due to heterogeneity in the primer sequences of other *Arcobacter* spp. [16]. Moreover, the *cdt* (*cdt*A, *cdt*B and *cdt*C) genes that play role as a virulence factor in promoting diseases by toxin-producing pathogens and genes that enable antibiotic resistance were not investigated in these studies [19, 51]. *A. faecis* and *A. lanthieri* are closely related to pathogenic species of *Arcobacter* (e.g., *A. butzleri* and *A. cryaerophilus*) [4, 13]; however, there has been no species- and gene-specific screening method developed that can efficiently be used to determine the pathogenicity, antibiotic resistance and toxicity which are all important factors when assessing the level of health risk an organism causes to humans or animals.

To our knowledge, this study reports the first *Arcobacter* species- and gene-specific mono- and multiplex PCR methods that have been developed and validated to detect and analyze the prevalence of several VAT genes in *A. faecis* and *A. lanthieri* individually. Although the initial time investment required to optimize species- or gene-specific mPCR assay was significantly more than that of the optimization of a monoplex assay, the long-term benefits are greater than the short-term costs. The high throughput and rapid screening of field isolates of both species for the analyses of the VAT genes cannot be understated. The process of optimizing species- and gene-specific mPCR assays commenced with careful design of primer pairs that may not only amplify sequences specific to the target gene, but also do not interfere with the other primer pairs used in each assay. Moreover, the fragment size can distinguishably be observed on an agarose gel. In the first phase of the study, all primers were carefully designed coupled with *Ex*-*Taq* DNA polymerase and BSA to improve yield and intensity of amplified products in a mixture of variable concentration of primers and DNA. To avoid preferential amplification of the shorter amplicons, we optimized multiple factors such as the concentration of each primer and the extension time of PCR cycles that may affect an mPCR assay. Although the primer pairs designed by considering various parameters using primer design software, they do not show optimized results until manipulation of several parameters (ranging from primers, Taq DNA polymerase, buffer and DNA concentrations to addition of BSA at various concentrations, annealing temperature and time in the PCR protocols) were applied during optimization of each mPCR assay. Moreover, multiple primer pairs with small amplicon sizes and distinctive differentiation preferably with a minimum amplicon length difference of 50 bp between amplicons of multiple target genes were designed for an mPCR assay with the aim to apply these assays in a real-time PCR environment which made optimization of each single-tube mPCR assay more challenging. After optimization of the above mentioned parameters, specific and consistent amplification of all targeted VAT genes in the mono- and multiplex PCR combinations were successfully achieved where a total of 11 including three mono- and eight multiplex PCR-based assays were developed, optimized and validated for the identification and differentiation of six virulence (*irg*A, *mvi*N, *cad*F, *tyl*A, *cia*B and *pld*A), two antibiotic resistance [*tet*(O) and *tet*(W)] and two/ three cytolethal distending toxin (*cdt*A, *cdt*B and *cdt*C) genes in *A. faecis* and *A. lanthieri*, respectively*.* In the first step, the primer set for each gene was individually optimized and tested in *A. faecis* and *A. lanthieri* reference strains where positive amplification reactions of all targeted VAT genes with expected product sizes were observed. Based on the monoplex PCR assay results, eight mPCR assays were further developed using a combination of two or three VAT gene primer pairs. Each optimized mPCR assay gave results consistent and similar, in terms of size and intensity, to the monoplex PCR assay and effectively detected VAT genes by distinguishably producing 18 clear bands in eight mPCR assays on an agarose gel electrophoresis. Due to limitations in fragment size and cross-reaction with primer pairs of other VAT genes, *tet*(O) and tet(W) gene were used as monoplex PCR assays.

To demonstrate the future utility and asses the degree of pathogenicity, the developed mono- and multiplex PCR assays were further validated by testing *A. faecis* and *A. lanthieri* strains isolated from agricultural surface water and fecal samples. Of the total ten VAT genes, *cad*F, *irg*A, *tyl*A, *tet*(W) and *cdt*C genes were detected in all *A. faecis* field isolates compared to one isolate that did not show amplification to *cia*B, *pld*A, and *cdt*A genes; whereas, two isolates were negative for *mvi*N and *tet*(O) genes. On the other hand, one strain of *A. lanthieri* showed negative amplification for all 11 VAT genes compared to two strains that did not amplify *irg*A, *tyl*A, *tet*(O), *cdt*A and *cdt*C genes when field isolates were tested for evaluation and validation of developed assays. The results are in agreement with other studies where *cad*F, *cia*B, *mvi*N, *pld*A, and *tyl*A virulence genes were detected at a high frequency in *A. butzleri* and *A. skirrowii* strains suggesting that these genes are common within the genus *Arcobacter* [10, 11, 14–16, 32, 52]. A high prevalence of *cia*B gene suggests that *A. faecis* and *A. lanthieri* are able to easily internalize into epithelial cells in the gastrointestinal lining that contributes in mediating enteritis [18]. On the other hand, *irg*A gene was detected in *A. faecis* and *A. lanthieri* strains tested which is not in concordance with previous studies where *irg*A gene was detected at a low frequency in *Arcobacter* spp. [10, 11]. The key function of *irg*A *in vivo* is uptake of iron, which is an essential but often severely limited nutrient in animal hosts meaning that low iron concentrations are less likely to prevent the pathogen from infecting and colonizing the host. This gene plays a key role in causing urinary tract infection by uropathogenic *E. coli* [53, 54]. However, the prevalence of *irg*A was significantly higher than the frequency observed in the aforementioned studies. This could be due to geographical differences from where the sample was obtained, for instance the samples analyzed in this study were isolated in Canada while these studies contained strains isolated from other parts of the world. Interestingly, similar to *A. faecis*, *tet*(O) and *tet*(W) antibiotic resistance genes were also detected at a significantly high frequency in *A. lanthieri*. The results are also supported by the previous study [31, 48] where tetracycline resistance in *A. cryaerophilus* was observed, suggesting that tetracycline resistance is more frequent in the genus *Arcobacter* than reported. Other than *Arcobacter* and *Campylobacter* spp., *tet*(O) and *tet*(W) genes were detected more significantly than other *tet*-genes [e.g., *tet*(B) and *tet*(C)] in commensal bacteria isolated from fecal and water samples [55]. Interestingly, similar to our optimized PCR assays where ≥93 *A. faecis* and ≥80% *A. lanthieri* isolates showed positive amplification for *tet*(O) and *tet*(W) genes, the phenotypic resistance to tetracycline was also observed at a significantly high (97%) rate in both species. These results suggest that when these species are clinically identified for tetracycline resistance, genotypic screening should be performed for the presence of *tet* genes since rapid, simple and reliable methods for antibiotic susceptibility are important for appropriate therapy. Moreover, identification and determination of the antimicrobial susceptibility test that requires a minimum of 3-days compared to the developed PCR assays that can be done in a few hours, therefore, the developed PCR assays can be used for confirmation of the results obtained by conventional phenotypic methods when necessarily required.

In contrary to the previous studies [19, 22], which reported the distinct absence of *cdt* genes in arcobacters and in whole genome sequence of *A. butzleri*, *cdt*A and *cdt*C genes were identified in *A. faecis* genome sequence where PCR assays detected these genes at high frequency (*cdt*A 93% and *cdt*C 100%) in field isolates. Interestingly, *cdt*B gene was not identified in the genome sequence of the *A. faecis* reference strain. This leads to a future research on genome sequencing of one of the field isolates that might contain all three *cdt* genes. If that is not the case, given bacterial ability to rapidly acquire new genes, it remains important to note that the field isolates currently possess both *cdt* genes that were investigated in this study. In contrast to *A. faecis*, all three *cdt* (*cdt*A, *cdt*B, *cdt*C) genes were found in the * A. lanthieri* genome sequence. The rates of detection of *cdt*A, *cdt*B and *cdt*C were significantly high where eight strains (80%) were shown positive for *cdt*A and *cdt*C genes compared to *cdt*B gene detected in nine strains (90%). The *cdt* genes were also detected at a variable frequency in *Campylobacter* spp. isolated from various sources [56–58]. The high frequency of prevalence of VAT genes and the results of the expression analysis of *cdt* genes where genes are functional in *A. faecis* and *A. lanthieri* strains indicate that these species of *Arcobacter* could potentially be pathogenic that can pose health risks to humans and animals.

One of the major advantages of these newly developed PCR assays is the significant specificity and sensitivity where each mono- or multiplex PCR assay showed specificity to their target species-specific genes at a least detection limit of 10 pg μL^-1^ DNA concentration (equivalent to 10^1^ CFU mL^-1^). The infectious dose for each pathogen varies; however, according to US EPA [59] guidelines, a pathogen can cause a disease when ingested at the rate of ≥10^3^ CFU mL^-1^. Although the infectious dose of *A. faecis* and *A. lanthieri* is not determined yet, the detection sensitivity of each assay is within the range that has been used for other bacterial pathogens and can be applied for developing standard for infectious dose and pathogenicity assessment of these species isolated from clinical and various environmental niches.

## Conclusion

The significance of investigating and detecting several VAT genes in *A. faecis* and *A. lanthieri* reference and field strains by developing, optimizing and validating eleven mono- and multiplex PCR assays described in this study would add useful knowledge to apply in the field of molecular microbiology. Moreover, the sensitivity, reproducibility and speed of the eight mPCR assays are enhanced due to the optimal application of several PCR parameters and addition of BSA that further enhanced the specificity of the assays by testing several other *Arcobacter* and bacterial species and strains as control. Overall, the rapidness and number of VAT genes tested in the developed PCR assays suggest that these novel PCR-based cost effective methods can be applied for rapid and possibly high throughput screening and assessment of pathogenic *A. faecis* and *A. lanthieri* strains in diagnostic laboratories and etiological/ epidemiological studies. Based on these results, further research is warranted to assess the role of these virulence factors on human or animal health by gene expression analysis or analysing the adhesive, invasive and toxicity capabilities in cell cultures using clinical and environmental isolates under various environmental conditions.

## Additional file

Additional file 1: **Table S1.** Validation of VAT PCR assays by sequencing of PCR products for *A. faecis* and *A. lanthieri* reference and field strains isolated from fecal and water samples. (DOCX 27 kb)

## Abbreviations

ASIA: Arcobacter Selective Isolation Agar
BSA: Bovine Serum Albumin
*cdt*: Cytolethal distending toxin
mPCR: Multiplex PCR
*tet*: Tetracycline resistance
VAGs: Virulence-associated genes
VAT: Virulence, antibiotic resistance and toxin
