# Supplementary material for: Novel virulence, antibiotic resistance and toxin gene-specific PCR-based assays for rapid pathogenicity assessment of Arcobacter faecis and Arcobacter lanthieri
Source: BMC Microbiol. 2019 Jan 11;19:11. doi: 10.1186/s12866-018-1357-7 (PMC6330389; doi:10.1186/s12866-018-1357-7)
Supplement: Supplementary file 1 — Table S1. Validation of VAT PCR assays by sequencing of PCR products for A. faecis and A. lanthieri reference and field strains isolated from fecal and water samples. (DOCX 27 kb) [file 12866_2018_1357_MOESM1_ESM.docx]

**Supplementary Table 1:** Validation of VAT PCR assay by sequencing of PCR products for *A. faecis* and *A. lanthieri* reference and field strains isolated from fecal and water samples

| **Strain** | **Species** | **Target gene** | **Source** | **Amplicon sequences** | **Product size (bp)** |
| --- | --- | --- | --- | --- | --- |
| LMG 28519 | *A. faecis* | *cad*F | Human septic tank | ATGGTGCATTCGGAAACTACGGTCTTGGTCTAAAATATCAATTAATTGATAGTTTAGCACTTAAATTTGATGTTAGACATTTAATTAACTTCAATTATGGAGATAATACAATGTTATATACATTAGGTTTAGCTGTTCCATTTGGAGAGAGAACAAAAGCTGCTCCAGAAGTAGTTCCTGCTCCAGCTCCAA | 192 |
| AF1055 | 1. *faecis* | *cad*F | Human septic tank | ATGGTGCATTCGGAAACTACGGTCTTGGTCTAAAATATCAATTAATTGATAGTTTAGCACTTAAATTTGATGTTAGACATTTAATTAACTTCAATTATGGAGATAATACAATGTTATATACATTAGGTTTAGCTGTTCCATTTGGAGAGAGAACAAAAGCTGCTCCAGAAGTAGTTCCTGCTCCAGCTCCAA | 192 |
| AW1119 | *A. faecis* | *cad*F | Agricultural water | ATGGTGCATTCGGAAACTACGGTCTTGGTCTAAAATATCAATTAATTGATAGTTTAGCACTTAAATTTGATGTTAGACATTTAATTAACTTCAATTATGGAGATAATACAATGTTATATACATTAGGTTTAGCTGTTCCATTTGGAGAGAGAACAAAAGCTGCTCCAGAAGTAGTTCCTGCTCCAGCTCCAA | 192 |
| LMG 28519 | *A. faecis* | *tyl*A | Human septic tank | TAGAAACAAAGCAAGTGAACTCATAAAATCCAATAAAGTAAAAATAGATGGAAAGATTATATCAAAAGCTTCATTTATAGTTGAAGAAGATATGAATATAGAGCTACTTGAAGAGG | 116 |
| AF1055 | *A. faecis* | *tyl*A | Human septic tank | TAGAAACAAAGCAAGTGAACTCATAAAATCCAATAAAGTAAAAATAGATGGAAAGATTATATCAAAAGCTTCATTTATAGTTGAAGAAGATATGAATATAGAGCTACTTGAAGAGG | 116 |
| AW1119 | *A. faecis* | *tyl*A | Agricultural water | TAGAAACAAAGCAAGTGAACTCATAAAATCCAATAAAGTAAAAATAGATGGAAAGATTATATCAAAAGCTTCATTTATAGTTGAAGAAGATATGAATATAGAGCTACTTGAAGAGG | 116 |
| LMG 28519 | 1. *faecis* | *cia*B | Human septic tank | AAGCAGTTGCCCTAGAGTGGGATATAAGAGTTACAAATCCAAAATTTGCACAAAATGACCATAGAGTAAATAAGATAAAAAGTGCATTTAGCAAAATTTATAGTAGTTTTGAGGCAAATACAAAATATAAAAAAATCTATGATTTTAGTTTCGCTTCACTTGATAAAGTTCAACTTTATGTTGGACGACCAGCACT | 196 |
| AF1055 | *A. faecis* | *cia*B | Human septic tank | AAGCAGTTGCCCTAGAGTGGGATATAAGAGTTACAAATCCAAAATTTGCACAAAATGACCATAGAGTAAATAAGATAAAAAGTGCATTTAGCAAAATTTATAGTAGTTTTGAGGCAAATACAAAATATAAAAAAATCTATGATTTTAGTTTCGCTTCACTTGATAAAGTTCAACTTTATGTTGGACGACCAGCACT | 196 |
| AW1119 | *A. faecis* | *cia*B | Agricultural water | AAGCAGTTGCCCTAGAGTGGGATATAAGAGTTACAAATCCAAAATTTGCACAAAATGACCATAGAGTAAATAAGATAAAAAGTGCATTTAGCAAAATTTATAGTAGTTTTGAGGCAAATACAAAATATAAAAAAATCTATGATTTTAGTTTCGCTTCACTtGATAAAGTTCAACTTTATGTTGGACGACCAGCACT | 196 |
| LMG 28519 | *A. faecis* | *pld*A | Human septic tank | GTGCTGCTGAATTTAACTGGACTTTCCCCCTTCCAGAATTTATGGCATCAAAAAATAGTTATGGGCTTTTTCAAATTTTTCATGGATATGGACAAAGTTTAATTGATTATGATAGAGAGCTTACAAATGTAGGAATAGGTGTTGC | 145 |
| AF1055 | *A. faecis* | *pld*A | Human septic tank | GTGCTGCTGAATTTAACTGGACTTTCCCCCTTCCAGAATTTATGGCATCAAAAAATAGTTATGGGCTTTTTCAAATTTTTCATGGATATGGACAAAGTTTAATTGATTATGATAGAGAGCTTACAAATGTAGGAATAGGTGTTGC | 145 |
| AW1119 | *A. faecis* | *pld*A | Agricultural water | GTGCTGCTGAATTTAACTGGACTTTCCCCCTTCCAGAATTTATGGCATCAAAAAATAGTTATGGGCTTTTTCAAATTTTTCATGGATATGGACAAAGTTTAATTGATTATGATAGAGAGCTTACAAATGTAGGAATAGGTGTTGC | 145 |
| LMG 28519 | *A. faecis* | *cdt*A | Human septic tank | TGTAGCCGATGAACTTAGTGAAGTAGAGTGTATTGAAGAATTTTTACAACAAAGATTTAGTAAAAAAATAAAAATAGTAAACCCAAAAATAGATAAAAAATCTACAATTATAAAAATTGCTTTAAATAACTGTGATGAACTTTTAAGACTTGAAAATATTAAAAATGAGAACTCTATTTATGAAGAACTTAAAGAACTATTTTCTCTAAGAACTACTCCAAATATAATAGAAGCTTTTGACAATTCTCATTTAATGGGACAAGCAACAGTTGGG | 274 |
| AF1055 | *A. faecis* | *cdt*A | Human septic tank | TGTAGCCGATGAACTTAGTGAAGTAGAGTGTATTGAAGAATTTTTACAACAAAGATTTAGTAAAAAAATAAAAATAGTAAACCCAAAAATAGATAAAAAATCTACAATTATAAAAATTGCTTTAAATAACTGTGATGAACTTTTAAGACTTGAAAATATTAAAAATGAGAACTCTATTTATGAAGAACTTAAAGAACTATTTTCTCTAAGAACTACTCCAAATATAATAGAAGCTTTTGACAATTCTCATTTAATGGGACAAGCAACAGTTGGG | 274 |
| AW1119 | *A. faecis* | *cdt*A | Agricultural water | TGTAGCCGATGAACTTAGTGAAGTAGAGAGTATTGAAGAGTTTTTACAACAAAGATTTAGTAAAAAAATAAAAATAGTAAATCCAAAAATAGATAAAAAAGCTACGATTATAAAAATTGCTTTAAATAACTGTGATGAGCTTTTAAGACTTGAAAATATTAAAAATGAAAACTCTATTTATGAAGAACTTAAAGAGCTATTCTCTTTAAAAACTACTCCAAATCTAATAGAAGCTTTTGACAATTCTCATTTAATGGGACAAGCAACAGTTGGG | 274 |
| LMG 28519 | *A. faecis* | *irg*A | Human septic tank | CTGGACAGTATGAAGGAAACCCTTTAAATGATATTTCTAAACATATGTTTAATGCGGGACTTGATTGGGATGTAACTTCTAAATGGTTGTTATGGACTCAAGCAA | 105 |
| AF1055 | *A. faecis* | *irg*A | Human septic tank | CTGGACAGTATGAAGGAAACCCTTTAAATGATATTTCTAAACATATGTTTAATGCGGGACTTGATTGGGATGTAACTTCTAAATGGTTGTTATGGACTCAAGCAA | 105 |
| AW1119 | *A. faecis* | *irg*A | Agricultural water | CTGGACAGTATGAAGGAAACCCTTTAAATGATATTTCTAAACATATGTTTAATGCGGGACTTGATTGGGATGTAACTTCTAAATGGTTGTTATGGACTCAAGCAA | 105 |
| LMG 28519 | *A. faecis* | *mvi*N | Human septic tank | TTCTTTGCAGCAACATTGGGCTCTTCAACAATGCATATTTCAGCATTTATTGATACTTGGTTGGCATCTTTTTTAATAAGTGGTTCAATTTCATATCTATATTATGCAAATAGAGTTTTTCAACTTCCACTAGCAATTTTTGCAATAGCAACATCAATTGCCCTATTTCCTATGGTAGCA | 180 |
| AF1055 | *A. faecis* | *mvi*N | Human septic tank | TTCTTTGCAGCAACATTGGGCTCTTCAACAATGCATATTTCAGCATTTATTGATACTTGGTTGGCATCTTTTTTAATAAGTGGTTCAATTTCATATCTATATTATGCAAATAGAGTTTTTCAACTTCCACTAGCAATTTTTGCAATAGCAACATCAATTGCCCTATTTCCTATGGTAGCA | 180 |
| AW1119 | *A. faecis* | *mvi*N | Agricultural water | TTCTTTGCAGCAACATTGGGCTCTTCAACAATGCATATTTCAGCATTTATTGATACTTGGTTGGCATCTTTTTTAATAAGTGGTTCAATTTCATATCTATATTATGCAAATAGAGTTTTTCAACTTCCACTAGCAATTTTTGCAATAGCAACATCAATTGCCCTATTTCCTATGGTAGCA | 180 |
| LMG 28519 | *A. faecis* | *tet*(O) | Human septic tank | GGAGGGGTTCAACCACAAAGTGAAACTGTTTGGAGACAAGCAAATAAGTATGGAGTCCAAGAATTATCTATGTAAATAAAATGGATAG | 88 |
| AF1055 | *A. faecis* | *tet*(O) | Human septic tank | GGAGGGGTTCAACCACAAAGTGAAACTGTTTGGAGACAAGCAAATAAGTATGGAGTCCAAGAATTATCTATGTAAATAAAATGGATAG | 88 |
| AW1119 | *A. faecis* | *tet*(O) | Agricultural water | GGAGGGGTTCAACCACAAAGTGAAACTGTTTGGAGACAAGCAAATAAGTATGGAGTCCAAGAATTATCTATGTAAATAAAATGGATAG | 88 |
| LMG 28519 | *A. faecis* | *tet*(W) | Human septic tank | ACATCATTCATACTCCAGGTCACGTTGACTTTACTATTGAAGTTGAGAGATCTATGAGGGTTCTTGATGGTGCTGTTGCTGTACCTTGTTCAGTTGGACCGGTTCAACCACAAAGTGAAA | 120 |
| AF1055 | *A. faecis* | *tet*(W) | Human septic tank | ACATCATTCATACTCCAGGTCACGTTGACTTTACTATTGAAGTTGAGAGATCTATGAGGGTTCTTGATGGTGCTGTTGCTGTACCTTGTTCAGTTGGACCGGTTCAACCACAAAGTGAAA | 120 |
| AW1119 | *A. faecis* | *tet*(W) | Agricultural water | ACATCATTCATACTCCAGGTCACGTTGACTTTACTATTGAAGTTGAGAGATCTATGAGGGTTCTTGATGGTGCTGTTGCTGTACCTTGTTCAGTTGGACCGGTTCAACCACAAAGTGAAA | 120 |
| LMG 28519 | *A. faecis* | *cdt*C | Human septic tank | AAGCAGAGGGTGAAATAGCCTTTGTTTTAAAAGAAGATTTAATGGGACCAGGTGTTACAACAGCTGATGTTTTAAGAGCTACAAAGTTTGTAATGCCTTGTTTTGAAATAGTTGATTCAAGAATTAAAGATTGGAAAATAAAAATTCAAGATACTGTTGCAGATAATGCCTCTTGTGGTTATATAGTATTTGGTGGACAGTTTGCTAATC | 210 |
| AF1055 | *A. faecis* | *cdt*C | Human septic tank | AAGCAGAGGGTGAAATAGCCTTTGTTTTAAAAGAAGATTTAATGGGACCAGGTGTTACAACAGCTGATGTTTTAAGAGCTACAAAGTTTGTAATGCCTTGTTTTGAAATAGTTGATTCAAGAATTAAAGATTGGAAAATAAAAATTCAAGATACTGTTGCAGATAATGCCTCTTGTGGTTATATAGTATTTGGTGGACAGTTTGCTAATC | 210 |
| AW1119 | *A. faecis* | *cdt*C | Agricultural water | AAGCAGAGGGTGAAATAGCCTTTGTTTTAAAAGAAGATTTAATGGGACCAGGTGTTACAACAGCTGATGTTTTAAGAGCTACAAAGTTTGTAATGCCTTGTTTTGAAATAGTTGATTCAAGAATTAAAGATTGGAAAATAAAAATTCAAGATACTGTTGCAGATAATGCCTCTTGTGGTTATATAGTATTTGGTGGACAGTTTGCTAATC | 210 |
| LMG 28516 | *A. lanthieri* | *cad*F | Pig manure storage tank | TCCAACTCCAGTTGCTGCTCCATTAGATAGCGATGGTGATGGTGTAATTGATGAATTAGATCAATGTCCAAATACTCCAAAAGGTGCAAAAGTTGATTCAGTTGGTTGTATTACATTAATTAACTTAAATGTTAACTTTGATACAGATAAATCTGATATTAAAGATGTATATAATACAAGAATTCACGAATTTGCTGAAGTTATGAAAACAGATAAAAAATTGAAAGCTGACATCGAAGGACA | 243 |
| AF0641 | *A. lanthieri* | *cad*F | Wastewater | TCCAACTCCAGTTGCTGCTCCATTAGATAGCGATGGTGATGGTGTAATTGATGAATTAGATCAATGTCCAAATACTCCAAAAGGTGCAAAAGTTGATTCAGTTGGTTGTATTACATTAATTAACTTAAATGTTAACTTTGATACAGATAAATCTGATATTAAAGATGTATATAATACAAGAATTCACGAATTTGCTGAAGTTATGAAAACAGATAAAAAATTGAAAGCTGACATCGAAGGACA | 243 |
| AW0110 | *A. lanthieri* | *cad*F | Agricultural water | TCCAACTCCAGTTGCTGCTCCATTAGATAGCGATGGTGATGGTGTAATTGATGAATTAGATCAATGCCCAAATACTCCAAAAGGTGCAAAAGTTGATTCAGTTGGTTGTATTACATTAATTAACTTAAATGTTAACTTTGATACAGATAAATCTGATATTAAAGATGTATATAATACAAGAATTCATGAATTTGCTGAAGTTATGAAAACAGATAAAAAATTGAAAGCTGACATCGAAGGACA | 243 |
| LMG 28516 | *A. lanthieri* | *irg*A | Pig manure storage tank | AGAGCTGTTGGTTGGGATGGTATTCTTTCAATGGGAACGATTACAGGAAAACAAAAGAATTCACCTTCTTATCAAAAATCAATAAATTATAATTATGACTTAACTCATGAATTAAAATATGATGAATTTTTAGTAAATTCATATTTAACTTATGAAAAATCAGAGAACCCTACAAGAGCAAATGCA | 186 |
| AF0641 | *A. lanthieri* | *irg*A | Wastewater | AGAGCTGTTGGTTGGGATGGTATTCTTTCAATGGGAACGATTACAGGAAAACAAAAGAATTCACCTTCTTATCAAAAATCAATAAATTATAATTATGACTTAACTCATGAATTAAAATATGATGAATTTTTAGTAAATTCATATTtAACTTATGAAAAATCAGAGAACCCTACAAGAGCAAATGCA | 186 |
| AW0110 | *A. lanthieri* | *irg*A | Agricultural water | AGAGCTGTTGGTTGGGATGGTATTCTTTCAATGGGAACGATTACAGGAAAACAAAAGAATTCACCTTCTTATCAAAAATCAATAAATTATAATTATGACTTAACTCATGAATTAAAATATGATGAATTTTTAGTAAATTCATATTTAACTTATGAAAAATCAGAGAACCCTACAAGAGCAAATGCA | 186 |
| LMG 28516 | *A. lanthieri* | *cdt*B | Pig manure storage tank | GCAAAAGGTGATTGGGCTCCAATTCATCCAAAAACATTTGAATCTAAACTTGTAAAAAATGTTCATGTAATAGGAGATGCTTCAATTGCTCAACCTATGCCAAAATCAGCATTTAGTGCAAGTACACAAGGAAAAGTTGTAGCTTTACAAATTGCAAGATTTTTAAAAGGTCAAGAGCCTATTAATCCTCCAAAACTCGCTAACACATGCTATAGTTTATTAAATCCTAACTATGGTATATCTATTGCAGCTGTGTATAATGCACATGATGATGTAATAGAGAGTGTTCAAGGAGCTGGAGGA | 303 |
| AF0641 | *A. lanthieri* | *cdt*B | Wastewater | GCAAAAGGTGATTGGGCTCCAATTCATCCAAAAACATTTGAATCTAAACTTGTAAAAAATGTTCATGTAATAGGAGATGCTTCAATTGCTCAACCTATGCCAAAATCAGCATTTAGTGCAAGTACACAAGGAAAAGTTGTAGCTTTACAAATTGCAAGATTTTTAAAAGGTCAAGAGCCTATTAATCCTCCAAAACTCGCTAACACATGCTATAGTTTATTAAATCCTAACTATGGTATATCTATTGCAGCTGTGTATAATGCACATGATGATGTAATAGAGAGTGTTCAAGGAGCTGGAGGA | 303 |
| AW0110 | *A. lanthieri* | *cdt*B | Agricultural water | GCAAAAGGTGATTGGGCTCCAATTCATCCAAAAACATTTGAATCAAAACTTGTAAAAAATGTTCATGTAATAGGAGACGCTTCAATTGCTCAACCTATGCCAAAATCAGCATTTAGTGCAAGTACACAAGGAAAAGTTGTAGCTTTACAAATTGCAAGATTTTTAAAAGGTCAAGAACCTATTAATCCTCCAAAACTTGCTAATACTTGCTATAGCTTACTAAACCCTAACTATGGTATATCTATTGCAGCTGTATATAATGCACATGATGATGTAATAGAGAGTGTTCAAGGAGCTGGAGGA | 303 |
| LMG 28516 | *A. lanthieri* | *cia*B | Pig manure storage tank | GATAGATGCTATTCTGCTCTTGTAAAAGAAAAAGATAGATATACATCAAAAGCTTACATAAAAGTTTTCAAAAAAGAAGTTACTAAAGTTGTAGATGCACTTGAAGAGTTTGCAGATAAACTAATAGAACTGGAAGATGAAATATATAATCAAAAATGGGAATATATAGCATACATACAGAGTTTAATAGTAGCATTTAGTGAAGAT | 207 |
| AF0641 | *A. lanthieri* | *cia*B | Wastewater | GATAGATGCTATTCTGCTCTTGTAAAAGAAAAAGATAGATATACATCAAAAGCTTACATAAAAGTTTTCAAAAAAGAAGTTACTAAAGTTGTAGATGCACTTGAAGAGTTTGCAGATAAACTAATAGAACTGGAAGATGAAATATATAATCAAAAATGGGAATATATAGCATACATACAGAGTTTAATAGTAGCATTTAGTGAAGAT | 207 |
| AW0110 | *A. lanthieri* | *cia*B | Agricultural water | GATAGATGCTATTCTGCTCTTGTAAAAGAAAAAGACTCATATACTTCAAAAGCTTATATAAAAGCTTTCAAAAAAGAAGTTACAGAAGTTGTAGATGCACTTGAAGAATTTGCAGATAAACTAATAGAACTTGAAGATGAAATATATAATCAAAAATGGGAATATATAGCATATATTCAAAGTTTAATAGTAGCATTTAGTGAAGAT | 207 |
| LMG 28516 | *A. lanthieri* | *tyl*A | Pig manure storage tank | GACATTGTAACATGTGATGTATCTTTTATATCTATTTTAAATATTATAAATGCTATAAATTCACTCCAATTTAAAGAAATTGTAATTCTTTTTAAACCACAATTTGAAGTGGGAACAAATGTAAA | 125 |
| AF0641 | *A. lanthieri* | *tyl*A | Wastewater | GACATTGTAACATGTGATGTATCTTTTATATCTATTTTAAATATTATAAATGCTATAAATTCACTCCAATTTAAAGAAATTGTAATTCTTTTtAAACCACAATTTGAAGTGGGAACAAATGTAAA | 125 |
| AW0110 | *A. lanthieri* | *tyl*A | Agricultural water | GACATTGTAACATGTGATGTATCTTTTATATCTATTTTAAATATTATTAATGCTATAAATTCACTCCAATTTAAAGAAATTGTAATTCTTTTTAAACCACAATTtGAAGTGGGAACAAATGTAAA | 125 |
| LMG 28516 | *A. lanthieri* | *mvi*N | Pig manure storage tank | ACCTTTGGTTCTTCAACTTTACATATTTCTGCTTTTATAGATACATGGTTAGCATCATTTTTAGTAAGTGGGTCAATATCATACTTATATTATGCAAATAGAGTTTTTCAGCTACCATTAGCAATTTTTGCAATAGCAACTTCAATAGCCCTATTTCCTATGGTAGCACG | 170 |
| AF0641 | *A. lanthieri* | *mvi*N | Wastewater | ACCTTTGGTTCTTCAACTTTACATATTTCTGCTTTTATAGATACATGGTTAGCATCATTTTTAGTAAGTGGGTCAATATCATACTTATATTATGCAAATAGAGTTTTTCAGCTACCATTAGCAATTTTTGCAATAGCAACTTCAATAGCCCTATTTCCTATGGTAGCACG | 170 |
| AW0110 | *A. lanthieri* | *mvi*N | Agricultural water | ACCTTTGGTTCTTCAACTTTACATATTTCTGCTTTTATTGATACTTGGTTAGCTTCATTTTTAGTAAGTGGTTCAATCTCATACTTATATTATGCAAATAGAGTTTTTCAGCTACCACTAGCAATTTTTGCAATAGCAACTTCAATAGCCCTATTTCCTATGGTAGCACG | 170 |
| LMG 28516 | *A. lanthieri* | *cdt*A | Pig manure storage tank | CAGGAATAGATCTCGCTACAAATGAAGATATTGATATTTTTGCAATAAACTCAAATAATAAAAAGGCTGTGATTGTAAGGATGTTTTTAAGAGATGGAAAACTTACCTCTTCAAATTATGATTTTATAAAAATAAATGATGATTTAGAATTTGATTATGAAGAAGCATATAAAAGAGCAATTATAAACTACTATGACAATGAACTTCCTCTTCTACCAAA | 220 |
| AF0641 | *A. lanthieri* | *cdt*A | Wastewater | CAGGAATAGATCTCGCTACAAATGAAGATATTGATATTTTTGCAATAAACTCAAATAATAAAAAGGCTGTGATTGTAAGGATGTTTTTAAGAGATGGAAAACTTACCTCTTCAAATTATGATTTTATAAAAATAAATGATGATTTAGAATTTGATTATGAAGAAGCATATAAAAGAGCAATTATAAACTACTATGACAATGAACTTCCTCTTCTACCAAA | 220 |
| AW0110 | *A. lanthieri* | *cdt*A | Agricultural water | CAGGAATAGATCTCGCTACAAATGAAGATATTGATATTTTTGCAATAAACTCAAATAATAAAAAGGCTGTGATTGTAAGGATGTTTTTAAGAGATGGAAAACTTACCTCTTCAAATTATGATTTTATAAAAATAAATGATGATTTAGAATTTGATTATGAAGAAGCATATAAAAGAGCAATTATAAACTACTATGACAATGAACTTCCTCTTCTACCAAA | 220 |
| LMG 28516 | *A. lanthieri* | *pld*A | Pig manure storage tank | TGCTCCATTTAGAGAAACTAACTATGAACCAGAATTATTTGTACAAATTCCACATAATAATGAACATTTAAAATTATCTAAAGTATCACTTATGCATTTTTCTAATGGTAAAGATGGTGAAGAATCTCGTTC | 132 |
| AF0641 | *A. lanthieri* | *pld*A | Wastewater | TGCTCCATTTAGAGAAACTAACTATGAACCAGAATTATTTGTACAAATTCCACATAATAATGAACATTTAAAATTATCTAAAGTATCACTTATGCATTTTTCTAATGGTAAAGATGGTGAAGAATCTCGTTC | 132 |
| AW0110 | *A. lanthieri* | *pld*A | Agricultural water | TGCTCCATTTAGAGAAACTAACTATGAACCAGAATTATTTGTACAAGTTCCACATAATAATGAACATTTAAAATTATCTAAAGTATCTCTTATGCATTTTtCTAATGGTAAAGATGGTGAAGAATCTCGTTC | 132 |
| LMG 28516 | *A. lanthieri* | *cdt*C | Pig manure storage tank | GATGAATCCACCAGAAATAGAGAAGTTTGAAAATATAACAAATCCTTTTGGAATAATCAATGCTTTATCAAATATTAAAAAAATAGAGAATAATAGACAACAATTTACTGCTTTAAAATTACAAATAGAAAATACTTTTCAAACTTTAGAAGAAGAGTTAAATAGTTATAAAGAACTTTATACTCTTGATCCCAAA | 196 |
| AF0641 | *A. lanthieri* | *cdt*C | Wastewater | GATGAATCCACCAGAAATAGAGAAGTTTGAAAATATAACAAATCCTTTTGGAATAATCAATGCTTTATCAAATATTAAAAAAATAGAGAATAATAGACAACAATTTACTGCTTTAAAATTACAAATAGAAAATACTTTTCAAACTTTAGAAGAAGAGTTAAATAGTtATAAAGAACTTTATACTCTTGATCCCAAA | 196 |
| AW0110 | *A. lanthieri* | *cdt*C | Agricultural water | GATGAATCCACCAGAAATAGAGAAGTTTGAAAATATAACAAATCCTTTTGGAATAATCAATGCTTTATCAAATATTAAAAAAATAGAGAATAATAGACAACAATTTACTGCTTTAAAATTACAAATAGAAAATACTTTTCAAACTTTAGAAGAAGAGTTAAATAGTTATAAAGAACTTTATACTCTTGATCCCAAA | 196 |
| LMG 28516 | *A. lanthieri* | *tet*(O) | Pig manure storage tank | TCATACACTTATACACTTGATGCACAAGCTGGAGAGATGTATAAAATTGAAGATATTCCAGCTGATTTAGTTGATGTTGCAAATGAATATAGAG | 94 |
| AF0641 | *A. lanthieri* | *tet*(O) | Wastewater | TCATACACTTATACACTTGATGCACAAGCTGGAGAGATGTATAAAATTGAAGATATTCCAGCTGATTTAGTTGATGTTGCAAATGAATATAGAG | 94 |
| AW0110 | *A. lanthieri* | *tet*(O) | Agricultural water | TCATACACTTATACACtTGATGCACAAGCTGGAGAGATGTATAAAATTGAAGATATTCCAGCTGATTTAGTTGATGTTGCAAATGAATATAGAG | 94 |
| LMG 28516 | *A. lanthieri* | *tet*(W) | Pig manure storage tank | AATCATACACTTATACACTTGATGCACAAGCTGGAGAGATGTATAAAATTGAAGATATTCCAGCTGATTTAGTTGATGTTGCAAATGAATATAGAGAAAAACTTGTTGAAGCTGCTGCTGAATCAAGTGATGAGCTAATGGATAAATATCTTGGTGGTGAAGAATTAACAGAAGAAGAGATAGTTGAAGGAATTAAA | 197 |
| AF0641 | *A. lanthieri* | *tet*(W) | Wastewater | AATCATACMCTTATACACTTGATGCACAAGCTGGAGAGATGTATAAAATTGAAGATATTCCAGCTGATTTAGTTGATGTTGCAAATGAATATAGAGAAAAACTTGTTGAAGCTGCTGCTGAATCAAGTGATGAGCTAATGGATAAATATCTTGGTGGTGAAGAATTAACAGAAGAAGAGATAGTTGAAGGAATTAAA | 197 |
| AW0110 | *A. lanthieri* | *tet*(W) | Agricultural water | AATCATACACTTATACACTTGATGCACAAGCTGGAGAAATGTATAAAATTGAAGATATCCCAGCAGATTTAGAAGATGTTGTAGCTGAATATAGAGAAAAACTAATTGAAGCTGCTGCTGAATCAAGTGATGAGTTAATGGATAAATATCTTGGTGGAGAAGAATTAACAGAAGAAGAGATAGTTGAAGGAATTAAA | 197 |
